# Exon-focused targeted oligonucleotide microarray design increases detection of clinically relevant variants across multiple NHS genomic centres

**DOI:** 10.1038/s41525-020-0136-1

**Published:** 2020-07-21

**Authors:** Jana Jezkova, Jade Heath, Angharad Williams, Deborah Barrell, Jessica Norton, Morag N. Collinson, Sarah J. Beal, Sian Corrin, Sian Morgan

**Affiliations:** 1 All Wales Medical Genomics Service, Cardiff and Vale University Health Board, NHS Wales, Cardiff, UK; 2 Wessex Regional Genetics Laboratory, Salisbury NHS Foundation Trust, Salisbury District Hospital, Salisbury, UK; 3 Bristol Genetics Laboratory, North Bristol NHS Trust, Bristol, UK

**Keywords:** Genetic testing, Medical genomics, Genetic testing, Neurodevelopmental disorders, Neurodevelopmental disorders

## Abstract

In recent years, chromosomal microarrays have been widely adopted by clinical diagnostic laboratories for postnatal constitutional genome analysis and have been recommended as the first-line test for patients with intellectual disability, developmental delay, autism and/or congenital abnormalities. Traditionally, array platforms have been designed with probes evenly spaced throughout the genome and increased probe density in regions associated with specific disorders with a resolution at the level of whole genes or multiple exons. However, this level of resolution often cannot detect pathogenic intragenic deletions or duplications, which represent a significant disease-causing mechanism. Therefore, new high-resolution oligonucleotide comparative genomic hybridisation arrays (oligo-array CGH) have been developed with probes targeting single exons of disease relevant genes. Here we present a retrospective study on 27,756 patient samples from a consortium of state-funded diagnostic UK genomic centres assayed by either oligo-array CGH of a traditional design (Cytosure ISCA v2) or by an oligo-array CGH with enhanced exon-level coverage of genes associated with developmental disorders (CytoSure Constitutional v3). The new targeted design used in Cytosure v3 array has been designed to capture intragenic aberrations that would have been missed on the v2 array. To assess the relative performance of the two array designs, data on a subset of samples (*n* = 19,675), generated only by laboratories using both array designs, were compared. Our results demonstrate that the new high-density exon-focused targeted array design that uses updated information from large scale genomic studies is a powerful tool for detection of intragenic deletions and duplications that leads to a significant improvement in diagnostic yield.

## Introduction

Over the last decade, the implementation of comparative genomic hybridisation arrays (array CGH) as a first-tier test for detecting chromosomal aberrations associated with developmental delay (DD), intellectual disability (ID), autism spectrum disorder, multiple congenital anomalies and other neurodevelopmental phenotypes has shown a marked improvement in diagnostic yields^1,2^. G-band karyotyping, the previously recommended test for patients with such phenotypes, provides a diagnostic yield of approximately 3%, while chromosomal microarray (CMA)-based testing has been shown to offer yields of 10–20%^1,2^.

Modern oligonucleotide-based array designs have typically focused on providing broad genome coverage with higher probe density in regions associated with specific disorders. More recently, newer array designs have been introduced that incorporate the findings of large-scale genomic studies (e.g., Deciphering Developmental Disorders study^3,4^) and the cumulative knowledge gained through the sharing of high-resolution, highly-curated genomic data in easily accessible public databases (e.g., ClinVar). A number of research groups have also advocated the use of exon-level probe coverage for specific genes, allowing further increases in diagnostic yield through the detection of smaller intragenic copy number variants (CNVs)^5,6^.

In order to understand the diagnostic impact of these array design strategies, we present here a large-scale in silico retrospective analysis of data derived from multiple state-funded UK based genomic centres who implemented the CytoSure Constitutional v3 array (CytoSure v3) for diagnostic testing of National Health Service (NHS) patients. The previous version, the CytoSure International Standards for Cytogenomic Arrays (ISCA) v2 (hereafter referred to as CytoSure v2), focuses on known disease-causing genes and has a “backbone” of probes across the entire genome. The new CytoSure v3 is a more focused, exon-centric oligo-array design that incorporates novel content from the DDD study and ClinVar database. Similar to the CytoSure v2 design, the v3 array has a reduced coverage of probes across uninformative regions to avoid detection of variants of unknown significance and incidental findings. We compare the performance of the CytoSure v3 array with that of the CytoSure v2 array; and also interrogate the entire post-natal proband dataset, providing further information on the type (i.e., duplication and deletion), size, frequency and chromosomal distribution of CNVs across the population tested.

## Results

### Total sample set and sample-level analysis

We analysed anonymised data from 27,756 postnatal blood samples obtained from four NHS laboratories. In total, 95,273 CNVs were identified, equating to an average of 3.43 CNVs per sample (Supplementary Table 1). This data set includes samples run on both the older v2 and newer v3 array. The distribution of duplications and deletions across individual chromosomes is shown in Supplementary Note 1 and the size ranges of CNVs detected are summarised in Supplementary Note 2.

**Fig. 1:**
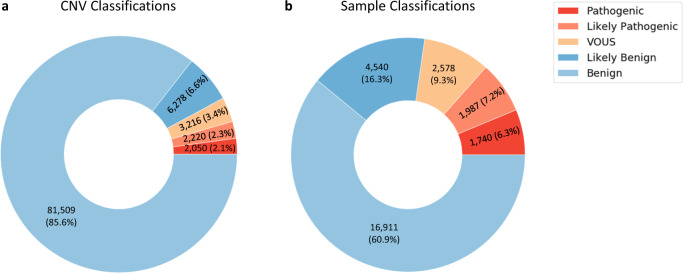
Total sample set and sample-level analysis. **a** Proportional classification of samples in the total data set—number of CNVs identified (percentage of total) in each classification bin (Benign, Likely Benign, VOUS, Likely Pathogenic and Pathogenic). **b** Sample-level Classifications—number of samples (percentage of total) classed as Benign, Likely Benign, VOUS, Likely Pathogenic or Pathogenic.

The majority of CNVs detected were identified as benign (Fig. 1). Viewing the results at the sample level, 6.27% of the samples had at least one CNV classified as pathogenic, and 7.16% of the samples had a call classified as likely pathogenic (Fig. 1b).

**Fig. 2:**
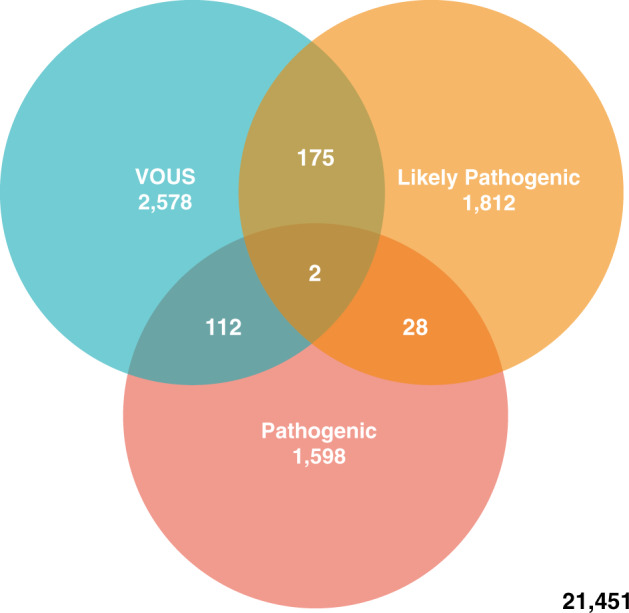
Venn diagram showing number of reportable samples by CNV classification. Regions of overlap indicate samples that contain more than one reportable CNV. Total number of samples: 27,756.

Collectively, all contributing laboratories classified 13.4% of all patient samples as containing a likely pathogenic or pathogenic CNV. Assuming that CNVs classified as variant of unknown significance (VOUS), likely pathogenic or pathogenic are reported, a reporting rate was calculated for all samples (Table 1). This indicates 22.72% of the patient samples tested possess a CNV detectable by array, which may be causative of a genetic disorder or require further investigation. In addition, 317 of the reportable cases exhibited more than one reportable CNV (Fig. 2), which equates to 1.14% of the total cohort.

**Table 1:** Sample reporting rate.

Total number of samples	Number of samples with ≥1 reportable CNV^a^ reported	Sample reporting rate
27,756	6,305	22.72%

^a^CNVs classified as either VOUS, likely pathogenic or pathogenic.

### Array comparison

This dataset includes two laboratories that transitioned from the v2 oligo-array to the v3 after in-house verification, and two laboratories that have only used the v3 array. In order to reduce the impact of inter-laboratory variation, only data from the two laboratories that ran both arrays were included in these analyses. In total, 19,675 samples were analysed, comprising 16,830 samples (85.54%) run on the CytoSure v2 array and 2845 samples (14.46%) run on the CytoSure v3 array.

**Fig. 3:**
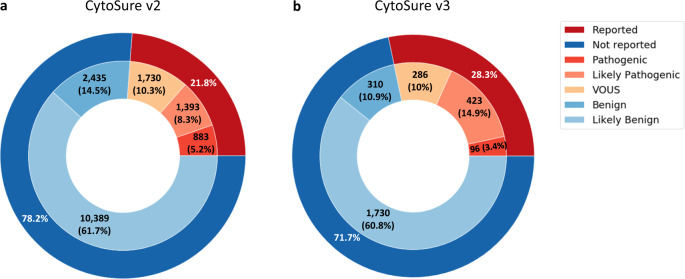
Proportional classification of samples by array design. **a** Traditional oligo-array design (CytoSure v2)—number of CNVs identified (percentage of total) in each classification bin (Benign, Likely Benign, VOUS, Likely Pathogenic and Pathogenic) and reporting rate. **b** Oligo-array CGH with enhanced exon-level coverage of genes identified as being important in developmental disorders from research projects (CytoSure v3)—number of samples (percentage of total) classed as Benign, Likely Benign, VOUS, Likely Pathogenic or Pathogenic and reporting rate.

When comparing sample classifications for both oligo-array designs, the largest difference is the higher proportion of samples classified as likely pathogenic using the CytoSure v3 array (Δ = +6.59%, Fig. 3).

In these contributing laboratories, an additional 4.72% of samples were found to have a likely pathogenic or pathogenic call when using the CytoSure v3 array in comparison to the CytoSure v2 array (v2 = 13.52, v3 = 18.24, Δ = +4.72). The proportion of samples classified as VOUS is comparable (~10%) for both oligo-array designs. Comparing the reporting rate between the two array designs shows a significant 4.49% increase (*p* value = 2.85e–07) for the CytoSure v3 array (Table 2). Whilst recognising the constraints of this study with regard to variability in samples and sample size, this increase indicates that the newer exon-focused array design provides a measurable improvement in diagnostic yield.

**Table 2:** Reporting rate of CytoSure v3 and v2 oligo-arrays.

	Total number of samples	Samples with likely pathogenic or pathogenic CNV	% samples with likely pathogenic or pathogenic CNV	Number of samples reported^a^	Reporting rate (%)
CytoSure v2	16,830	2,276	13.5	4,006	23.80
CytoSure v3	2,845	519	18.24	805	28.29

^a^Number of samples classified as either VOUS, likely pathogenic or pathogenic.

### Intragenic copy number changes detected by exon-focused array design

The CytoSure v3 array showed a significant increase in intragenic CNVs per sample when compared to the CytoSure v2 array (0.73 vs. 0.5; *p* value < 2.2e–16, Table 3).

**Table 3:** Number of intragenic CNVs and samples for each array design.

Arrays	# of intragenic CNVs	# samples	% of total samples	Intragenic CNVs/sample
CytoSure v2 8 × 60 K	8,560	16,830	50	0.5
CytoSure v3 8 × 60 K	7,930	10,926	73	0.73

Table 4 lists the 62 cases with pathogenic and likely pathogenic intragenic CNVs identified in our cohort by the CytoSure v3 array that would have been missed by the v2 array and shows that the newer exon-focused array design is a powerful tool for detection of intragenic imbalances, improving diagnostic yield. The dataset supporting this conclusion is included as Supplementary Data 1.

**Table 4:** Pathogenic and likely pathogenic intragenic CNVs identified by the CytoSure v3 oligo-array in 27,756 unique samples that would have been missed by the v2 oligo-array.

Gene	Disease	Inheritance	Cases (*n*)
ANKRD11	KBG syndrome (OMIM#148050)	AD	1
ARSE	Chondrodysplasia punctata, X-linked recessive (OMIM#302950)	XLR	1
ATP7A	Menkes disease (OMIM#309400), Occipital horn syndrome (OMIM#304150), Spinal muscular atrophy, distal, X-linked 3 (OMIM#300489),	XLR	2
AUTS2	Mental retardation, autosomal dominant 26 (OMIM#615834)	AD	6
BCAP31	Deafness, dystonia, and cerebral hypomyelination (OMIM#300475)	XLR	1
BRCA2	Fanconi anaemia, complementation group D1 (OMIM#605724), Wilms tumour (OMIM#194070)	AR, AD	1
CASK	FG syndrome 4 (OMIM#300422), Mental retardation and microcephaly with pontine and cerebellar hypoplasia (OMIM#300749), Mental retardation, with or without nystagmus (OMIM#300422)	XLD	2
CHD8	Autism, susceptibility to, 18 (OMIM# 615032)	AD	1
CTNNB1	Exudative vitreoretinopathy 7 (OMIM#617572), Mental retardation, autosomal dominant 19 (OMIM#615075)	AD	1
DDX3X	Mental retardation, X-linked 102 (OMIM#300958)	XLD, XLR	1
DEPDC5	Epilepsy, familial focal, with variable foci 1 (OMIM#604364)	AD	1
DMD	Duchenne muscular dystrophy (OMIM#310200)	XLR	2
EHMT1	Kleefstra syndrome 1 (OMIM#610253)	AD	1
FBN1	Acromicric dysplasia (OMIM#102370), Ectopia lentis, familial (OMIM#129600), Geleophysic dysplasia 2 (OMIM#614185), Marfan lipodystrophy syndrome (OMIM#616914), Marfan syndrome (OMIM#154700), MASS syndrome (OMIM#604308), Stiff skin syndrome(OMIM#184900), Weill-Marchesani syndrome 2, dominant (OMIM#608328)	AD	1
FHL1	Hemophagocytic lymphohistiocytosis, familial, 1 (OMIM#267700)	AR	2
FOXP1	Mental retardation with language impairment and with or without autistic features (OMIM#613670)	AD	1
GRIN2B	Epileptic encephalopathy, early infantile, 27 (OMIM#616139), Mental retardation, autosomal dominant 6 (OMIM#613970)	AD	1
HDAC8	Cornelia de Lange syndrome 5 (OMIM#300882)	XLD	1
HERC2	Mental retardation, autosomal recessive 38 (OMIM#615516)	AR	1
MBD5	Mental retardation, autosomal dominant 1 (OMIM#156200)	AD	8
MED13L	Mental retardation and distinctive facial features with or without cardiac defects (OMIM#616789), Transposition of the great arteries, dextro-looped 1 (OMIM#608808)	AD	5
MEF2C	Mental retardation, stereotypic movements, epilepsy, and/or cerebral malformations (OMIM#613443)	AD	2
NRXN1	Pitt-Hopkins-like syndrome 2 (OMIM#614325)	AR	1
NSDHL	CHILD syndrome (OMIM#308050), CK syndrome (OMIM#300831)	XLD, XLR	2
PAX2	Glomerulosclerosis, focal segmental, 7 (OMIM#616002), Papillorenal syndrome (OMIM#120330)	AD	1
PTHLH	Brachydactyly, type E2 (OMIM#613382)	AD	1
PTPN11	LEOPARD syndrome 1 (OMIM#151100), Metachondromatosis (OMIM#156250), Noonan syndrome 1 (OMIM#163950)	AD	2
RASA1	Capillary malformation-arteriovenous malformation 1 (OMIM#608354)	AD	1
RUNX1	Leukaemia, acute myeloid (OMIM#601626), Platelet disorder, familial, with associated myeloid malignancy (OMIM#601399)	AD	4
SATB2	Glass syndrome (OMIM#612313)	AD	1
SCN2A	Epileptic encephalopathy, early infantile, 11 (OMIM#613721), Seizures, benign familial infantile, 3 (OMIM#607745)	AD	1
SMARCA2	Nicolaides-Baraitser syndrome (OMIM#601358)	AD	1
STXBP1	Epileptic encephalopathy, early infantile, 4 (OMIM#612164)	AD	1
TCF4	Corneal dystrophy, Fuchs endothelial, 3 (OMIM#613267), Pitt-Hopkins syndrome (OMIM#610954)	AD	1
USP9X	Mental retardation, X-linked 99 (OMIM#300919), Mental retardation, X-linked 99, syndromic, female-restricted (OMIM#300968)	XLR, XLD	2

*AD* autosomal dominant, *AR* autosomal recessive, *XLR* X-linked recessive, *XLD* X-linked dominant.

## Discussion

In recent years, CMAs have been widely adopted by clinical diagnostic laboratories for postnatal constitutional genome analysis. Because of its higher detection rate compared to conventional karyotyping, CMAs have been recommended as the first-line test for postnatal cases with ID, DD, autism and/or congenital abnormalities^1,7^. A diagnostic array platform for this category of referrals should achieve a resolution of at least 200–400 kb^1,7^. There are several designs and array platforms available. Gene-targeted oligo arrays have a backbone of probes evenly spaced throughout the genome with increased density of probes at regions corresponding to genes of interest. This traditional design enables the identification of CNVs containing whole gene or multiple exons. However, this level of resolution often cannot detect pathogenic intragenic deletions or duplications, which represent a significant disease causing mechanism^8^. Therefore, new high-resolution array CGH have been developed with probes targeting single exons of disease relevant genes.

In this study, 27,756 unique patient samples were assayed by either oligo-array CGH of a traditional design (Cytosure v2) or by an oligo-array CGH with enhanced exon-level coverage of genes identified as being important in developmental disorders from research projects (Cytosure v3). In total, 95,273 CNV calls were identified with an average of 3.43 CNVs per sample. Pathogenic or likely Pathogenic CNVs were found in 13.43% of all patient samples. When comparing the two array designs, an additional 4.72% of samples were found to have a likely pathogenic or pathogenic call when using the CytoSure v3 array in comparison to the CytoSure v2 array. Overall, the diagnostic yield in our cohort was comparable with previous studies in patients with ID and DD, where the detection rate of pathogenic array CGH identified by array CGH ranged from 7 to 20%^1,9–15^.

The rate of reportable CNVs (VOUS, likely pathogenic or pathogenic) reached 22.72% with more than one reportable CNV identified in 5.07% of the reportable cases. As expected, the CytoSure v3 array showed a significant improvement in reporting rate (Δ = +4.49%, *P* = 2.85e–07) compared to the CytoSure v2 design. This is due to an increase detection of clinically significant CNVs classified as pathogenic or likely pathogenic while mitigating an increase in VOUS rate, indicating a well-balanced design of the new v3 array.

However, as CNVs were interpreted based on current knowledge at the time of the testing, the difference in the rate of reportable CNVs may also be partly explained by changes of classification of the imbalances over time. This may be due to the availability of new large datasets generated through genomic initiatives, such as the DDD study^4^, or additional information that has become available, such as data from family or functional studies. Careful, phenotype-driven selection of patients that are most likely to benefit from testing can also lead to improvement of diagnostic yield.

The CytoSure v3 array design has an increased exon-level coverage over clinically relevant regions. We hypothesised that we could identify more intragenic pathogenic CNVs with the v3 array design than with CytoSure v2. Similar to other studies^6,8,16^, the CytoSure v3 array detected a high proportion (73%) of intragenic CNVs and was able to call small likely pathogenic and pathogenic intragenic aberrations in known ID genes that would have been missed on the CytoSure v2 array (summarised in Table 4). These array findings were consistent with previous investigations, which detected intragenic CNVs in an overlapping set of genes identified in our study^16^.

The results of our study are limited by the use of different patient samples on the two array platforms. Comparison of the same patient samples using both array designs would provide a more rigorous data set; however, the cost of such an endeavour makes it unfeasible to achieve on a large sample size within the framework of an NHS laboratory performing routine diagnostic testing. In addition, we observed some cross-laboratory variability in classification of CNVs, highlighting the need for standardised clinical interpretation guidelines. The American College of Medical Genetics (ACMG) has recently issued new guidelines for the interpretation and reporting of CNVs in routine diagnostics^17^. Once ratified by ACGS, these guidelines should help to reduce the inconsistencies in clinical interpretation. One example of a conflict in interpretation from this study is single or multi-exon CNVs occurring in the JARID2 gene detected in five patients that were classified by the contributing laboratories as either Likely Benign, VUS or Likely Pathogenic. The difference in classification likely reflects historical guidelines and evidence available at the time of the original interpretation as recent studies suggest this is a benign polymorphism occurring at a high population frequency^16,18^.

Until NGS-based approaches progress to be able to reliably detect CNVs in a routine diagnostic setting, exon-focused CMAs will remain an important complementary tool to WES and WGS for analysis of both large and small genomic copy number imbalances^19–25^. Targeted arrays offer a stable, proven platform with simple data analysis and minimal demands on bioinformatics capacity, a common limiting factor for some laboratories when adopting NGS as a routine test to detect CNVs in a large cohort of patients. To ensure the continued targeting of the most disease-relevant regions, CMA design must be routinely updated to incorporate latest genomic information delivered by high-resolution analysis technologies and the findings of large genomic projects.

In conclusion, we report a measurable improvement in diagnostic yield in NHS patients assayed by an oligonucleotide array CGH with enhanced exon-level coverage of genes important for developmental disorder research (Cytosure v3) in a very large cohort from a consortium of state-funded diagnostic genomic centres in the UK. Our results also show that the new exon-focused array design provides a powerful tool for detection of small pathogenic intragenic deletions and duplications, providing a clinical answer for a proportion of patients that would have remained undiagnosed. At this time, targeted arrays offer a robust method of copy number analysis to supplement NGS based tests.

## Methods

### Data acquisition

Developing a curated dataset suitable for analysis required array processing, calling of CNVs, data acquisition, classification binning and data filtering. All samples and data were collected retrospectively from a cohort of patients with neurodevelopmental disorders who underwent routine phenotypic-driven diagnostic testing in the NHS setting. Since the aim of this study is to assess the performance of the CytoSure v3 array platform across multiple ISO 15189 UKAS accredited diagnostic genetic laboratories in a routine NHS diagnostic setting, the acquired data has been anonymised and contained no further clinical information (e.g., gender, phenotype, referral type and additional follow-up activities) in order to maintain patient confidentiality. All contributing laboratories performed their own technical verifications, including calculating performance parameters and running samples in parallel, to satisfy ISO 15189 requirements. For example summary of validation data see Supplementary Note 3.

Clinical consent was obtained for all samples prior to genetic testing. For our retrospective analysis, we used anonymised data from existing diagnostic samples for which formal ethical approval was not required. Our study did not involve prospective collection of samples or changing care from accepted standards for any of the patients. The study was approved by individual Trusts as a service evaluation and an approval from Information Governance in each Health Board was obtained to collect the anonymised data.

### Array processing

DNA were extracted from post-natal patient samples using laboratory-specific protocols. DNA samples were labelled using the CytoSure Genomic DNA Labelling Kit prior to running on the CytoSure ISCA v2 (8 × 60 k) array or CytoSure Constitutional v3 (8 × 60 k) array according to the manufacturer’s (Oxford Gene Technology, Oxford, UK) guidelines. Aberration detection was performed using CytoSure Interpret Software according to analysis protocols validated by each contributing laboratory.

### Calling algorithms

CNV detection within CytoSure Interpret Software is carried out using a circular binary segmentation algorithm in order to group probes into regions of equal copy number. Thresholds are applied based on log ratio and region size to determine which segments are significant and therefore represent a loss or a gain. These segments are hereby described as calls.

### Data acquisition

#### Classification binning

Each contributing laboratory deploys their own call classification structures. To allow consistency of data comparison, all participating laboratories were asked to ‘bin’ their classification structure into five standardised classifications (‘benign’, ‘likely benign’, ‘VOUS’, ‘likely pathogenic’ and ‘pathogenic’), which were subsequently used for all downstream analyses.

### Data curation

#### Total sample set

After filtering (see Supplementary Note 4), the final data set comprised 95,273 calls from 27,756 unique samples, hereafter referred to as CNVs to differentiate from unfiltered data, referred to as calls).

#### Sample-level analysis

In order to analyse data at the sample level, we collapsed all records such that there was only one CNV per sample, retaining the CNV with the most pathogenic classification. These records are hereafter described as samples.

#### Array comparison

To assess the relative performance of the exon-focused CytoSure v3 array against its less focussed predecessor, the CytoSure v2 array, clinical data generated using both array designs was compared. To reduce potential sources of variation (e.g., different clinical referral pathways and analysis workflows), only data from the two laboratories that had utilised both array designs were used in these analyses.

#### Statistical analysis

All statistical analyses were performed in R version v3.5.0. The Test of Equal or Given Proportions was used for all comparisons.

#### Reporting rate

The reporting rate was calculated based on the assumption that only samples with one or more CNV classified as VOUS, likely pathogenic or pathogenic are reported [Eq. (1)]. Although there are variations in inter-laboratory policies, for the most part VOUS are reported as such upon initial identification pending receipt and analysis of parental samples.

1 $$\frac{{{\mathrm{Number}}\,{\mathrm{of}}\,{\mathrm{samples}} \ge 1\,{\mathrm{Reportable}}\,{\mathrm{CNV}}}}{{{\mathrm{Total}}\,{\mathrm{number}}\,{\mathrm{of}}\,{\mathrm{samples}}}} \times 100.$$

### Reporting summary

Further information on experimental design is available in the Nature Research Reporting Summary linked to this article.

## Supplementary information

Supplementary Information

Reporting Summary

Supplementary Data 1

## Data Availability

The datasets generated and/or analysed during the current study are not publicly available to maintain patient confidentiality, but are available from the corresponding author on reasonable request. When consent for open-access was available, anonymised genotype and phenotype data was deposited in DECIPHER (https://decipher.sanger.ac.uk/) under the following project IDs: NHS-CAR, NHS-BWH, NHS-WSX and NHS-SMB and shared via the NHS consortium projects.
