# Supplementary material for: Exon-focused targeted oligonucleotide microarray design increases detection of clinically relevant variants across multiple NHS genomic centres
Source: NPJ Genom Med. 2020 Jul 21;5:28. doi: 10.1038/s41525-020-0136-1 (PMC7374691; doi:10.1038/s41525-020-0136-1)
Supplement: Supplementary file 1 — Supplementary Information [file 41525_2020_136_MOESM1_ESM.pdf]

SUPPLEMENTARY INFORMATION

Contents

Supplementary Table 1. Overview of CNVs and samples for each NHS laboratory……2

Supplementary Note 1. Chromosomal distribution of CNVs……………………………….………3

Supplementary Note 2. CNV sizes…………………………………………………………………..……4

Supplementary Note 3. Summary of verification of the OGT CytoSure Constitutional v3 array CGH………………………………………………………………………………………..………………5

Supplementary Note 4. Data acquisition and filtering……………………………..…….………7

Supplementary Data 1. Description………………………………..………………………………8

Laboratory	# CNVs		Total number of calls	% of total calls	# Samples		Total # samples	% of total samples	Calls/ sample v2	Calls/ sample v3	Total calls/ sample
	v2	v3			v2	v3					
Laboratory A	0	14,633	14,633	15.36	0	5,979	5,979	21.54	N/A	2.45	2.45
Laboratory B	39,531	4,708	44,239	46.43	11,097	1,459	12,556	45.24	3.56	3.23	3.52
Laboratory C	0	7,411	7,411	7.78	0	2,102	2,102	7.57	N/A	3.53	3.53
Laboratory D	23,900	5,090	28,990	30.43	5,733	1,386	7,119	25.65	4.17	3.67	4.07
			95,273				27,756				

Supplementary Table 1. Overview of CNVs and samples for each NHS laboratory.

Supplementary Note 2. Chromosomal distribution of CNVs.

Chromosomal distribution of CNVs

Analysing duplications and deletions detected across all samples for each chromosome reveals a number or chromosomes across the sample set with proportionately higher levels of CNV, in particular chromosomes X, Y and 8. The total number of duplications and deletions detected for all samples 108,039 and 74,309 observed respectively. The distribution of duplications and deletions across individual chromosomes is shown by Figure 2.1.

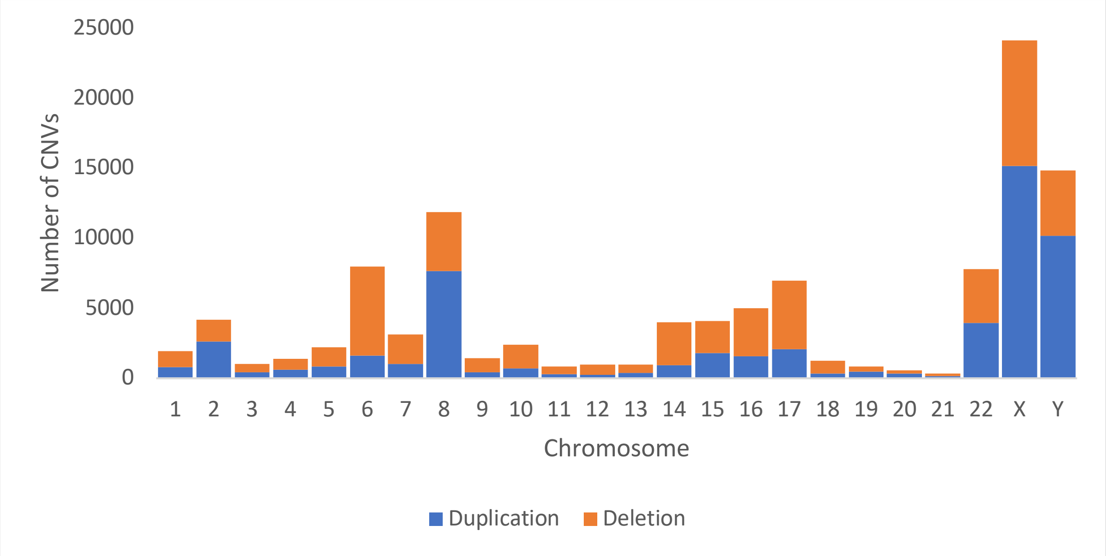

Figure 2.1. Total number of duplications and deletions for all samples by chromosome.

Supplementary Note 3. CNV sizes.

The number and size of CNVs detected using the two array designs were analysed for each classification (Figure 3.1) for the two labs that used the v2 and v3 arrays. The CNVs within the benign classification are typically smaller in size than those in more pathogenic classifications, culminating with a predominance of large CNVs in the pathogenic classification. This is to be expected, as the impact of large CNVs is more likely to interrupt 'normal' gene function, leading to a clinical phenotype.

Comparing CNV lengths across the different classifications between the two array designs reveals relatively concordant data, with the largest discrepancies evident in the VOUS and pathogenic classifications. In the VOUS classification, a larger proportion of smaller CNVs were detected by the CytoSure v3 array when compared to the CytoSure v2 array. In the pathogenic classification, there are markedly more 'larger' CNVs detected using the CytoSure v2 array than evident using the CytoSure v3 array.

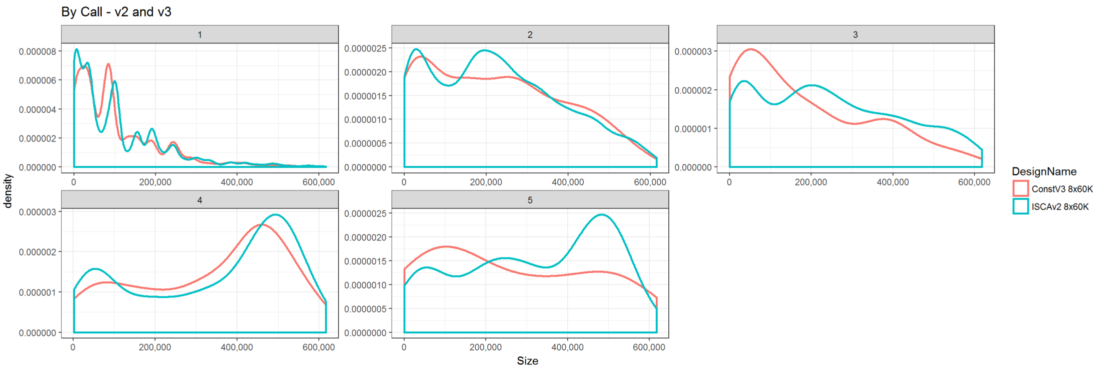

Figure 3.1. CNV density plots for each classification bin. 1= benign, 2=likely benign, 3=VOUS, 4=Likely Pathogenic, 5=Pathogenic.

Supplementary Note 5. Summary of verification of the OGT CytoSure Constitutional v3 array CGH, Wessex Regional Genetics Laboratory, Salisbury NHS Foundation Trust, Salisbury, UK

1. *Estimation of general sensitivity and specificity for array CGH testing of prenatal samples*

The data used in this validation includes only samples that were

- Analysed and found to be normal by array-CGH which was confirmed by parallel conventional karyotyping analysis
- Analysed and found to have an imbalance by array-CGH which was then confirmed by conventional karyotyping, FISH, MLPA or parental follow up.

Results:
In total, 131 prenatal samples have been analysed using array CGH.
Of these, 33 samples have shown to have imbalances on array CGH which have been confirmed either by conventional karyotyping, FISH, MLPA or parental array-CGH.
Of these, 98 samples have shown to have no imbalances on array CGH which have been confirmed by conventional karyotyping.

Estimates of accuracy and measures of uncertainty:
Sensitivity: >99% overall with 95% confidence interval (range of 89.32% to 100%)
Specificity: >99% overall with 95% confidence interval (range of 96.27% to 100%)

Overall Conclusion: Array-CGH is acceptable for analysis of prenatal samples.

2. *Verification of OGT microarray-CGH slide version 3 for diagnosis of chromosomal imbalances in referrals with developmental delay, congenital abnormalities and/or dysmorphism in the postnatal setting*

   2.1. Aim: To estimate intermediate precision of the array probes in a patient sample run on separate slides

Results:
SD for individual probes = 0.048
At Log2 Ratio value of -1, CV% = 4.8%
At Log2 Ratio value of 0.58, CV% = 8.3%

Conclusion: High precision across the dynamic range values.

   2.2. Aim: To calculate technical noise of array probes (around zero) in a self –self hybridisation where all ratios should be zero

Results:
Technical noise for normal call (2:2 allele ratio), exclusive of any biological variation
SD = 0.13
-2.62 to 0.262 (95%)

-0.344 to 0.344 (99%)

Conclusion: 99% range limits fall well within dynamic range, reducing false positive or outliers due to technical noise alone.

2.3. Aim: To assess the success rate of the new slide design, as compared to the current design

Results:
56 patient samples were processed and analysed. DLRS and waviness scores were extracted from the Cytosure Quality metric summary.
Success rate: 54/56 cases = 96%
Of the 2 failed patients, one was due to poor DNA quality and the other due to atmospheric Ozone. The original validation reported a success rate of 99% over 500 samples.

Conclusion: Success rate >95%

2.4. Aim: To verify whether the new slide design meets the key quality metrics

Results:
Average DLRS score: 0.125 (<0.2 = excellent)
Average Waviness score: 0.014 (<0.025 = excellent)

Conclusion: This is comparable to the 0.11 average DLRS reported in the original validation, and verifies that the new slide design is achieving the desired quality criteria.

2.5. Aim: To calculate accuracy and detection rate of the new slide design compared to the current design.

Results:
Array-CGH Success rate: 96% (data for 54/56)
Accuracy of detection of pathogenic known CNVs: 100% (27/27 patients)
Increased detection rate of clinically relevant CNVs: 10% (5/50 patients)

Conclusion: The new array-CGH slide design meets the required quality parameters and is acceptable for analysis of postnatal samples referred with developmental delay, congenital abnormalities and dysmorphism.
The results are comparable to those obtained from the previous slide design and the improved probe coverage of specific genomic regions means the detection rate for clinically relevant CNVs has improved.

3. Uncertainty of Measurement

Normalised log ratio values pulled from OGT software, descriptive statistics for non-parametric data generated for each sample. Variance data generated for each

sample type. Deming's regression analysis used to assess differences between variance of data set.

Uncertainty of Measurement – at probe level
Variance = 0.007
Median = 0.005

Interquartile range

0.25 limit	0.75 limit	IQR
-0.050472451	0.05933509	0.102062296

95% range

0.025 limit	0.975 limit	95% range
-0.165546549	0.164979837	0.312515432

Supplementary Note 4. Data acquisition and filtering.

Data was acquired, with the permission of the participating laboratories, using a plug-in (software component) for CytoSure Interpret Software. The data was retrieved from each laboratory's relational database in four separate files as detailed below.

CNV_CALLS file
Contains all calls in the database as a separate line item. Each line of the text file contains:

- Anonymised sample ID: A random unique string of characters, produced at runtime. No identifiable information is included in the dataset.
- Array barcode: Identifies the array used to make the call
- Chromosome of the call
- Start (max): Maximum genomic upstream position of the call
- Start (min): Minimum genomic upstream position of the call
- End (min): Minimum genomic downstream position of the call
- End (max): Maximum genomic downstream position of the call
- Mean log ratio: Average log ratio of all normalised log ratios of the probes that contributed to the call
- Number of probes: Number of probes contributing to the call
- Classification: Laboratory-defined classification (e.g., benign, pathogenic etc.)
- Analysis protocol name: Identifier of the CytoSure Interpret analysis protocol used

QC_METRICS file
Contains QC metrics of the array runs in the database, including:

- Array barcode
- QC metrics for the array (DLR spread, signal intensity, background noise, etc.)

PROTOCOLS file

Contains descriptions of every analysis protocol available in the software, including details such as:

- Number of probes required to make a call
- Segmentation algorithm used
- Thresholds used for defining a segment as loss or gain

ANONYMISED_SAMPLE_IDS
Contains mappings from the anonymised sample ID and any original sample ID's used by the lab. This file is not used in any analysis and is kept by the contributing lab, in order to match up any anonymised sample information if deemed appropriate.

DATA FILTERING
The initial data set obtained from the four participating laboratories comprised 31,314 unique, anonymised samples with a total of 182,348 calls. This study focuses on post-natal proband samples only and an initial filtering step was applied to exclude samples which fell outside of this criterion. These included samples run on alternative array formats (17 CNVs) and samples that were not compared against a known reference (i.e. sample-on-sample data; 4,038 CNVs).

83,020 calls classified by the laboratories as "N/A" were also removed from subsequent analyses. These calls comprise artefacts, which are known exceptions reflective of the array build, Y-chromosome calls and any unclassified calls (calls where no final classification has been assigned, e.g. on failed samples).

After filtering, the final data set comprised 95,273 calls from 27,756 unique samples, hereafter referred to as copy number variations (CNVs) to differentiate from unfiltered data, referred to as calls. This represents a reduction of 47.75% of the total calls and 11.36% of the samples.

Supplementary Data 1. Description

Full dataset containing pathogenic and likely pathogenic intragenic CNVs identified by the CytoSure v3 oligo-array in 27,756 unique samples that would have been missed by the v2 oligo-array.
